# Linking Physical Activity to Breast Cancer Risk via Inflammation, Part 1: The Effect of Physical Activity on Inflammation

**DOI:** 10.1158/1055-9965.EPI-22-0928

**Published:** 2023-03-03

**Authors:** Christopher T.V. Swain, Ann E. Drummond, Roger L. Milne, Dallas R. English, Kristy A. Brown, Makayla W.C. Lou, Leonessa Boing, Amy Bageley, Tina L. Skinner, Eline H. van Roekel, Melissa M. Moore, Tom R. Gaunt, Richard M. Martin, Sarah J. Lewis, Brigid M. Lynch

**Affiliations:** 1 Cancer Epidemiology Division, Cancer Council Victoria, Melbourne, Australia.; 2 Centre for Epidemiology and Biostatistics, Melbourne School of Population and Global Health, The University of Melbourne, Melbourne, Australia.; 3 Precision Medicine, School of Clinical Sciences at Monash Health, Monash University, Melbourne, Australia.; 4 Department of Medicine, Weill Cornell Medicine, New York, New York.; 5 Laboratory of Research in Leisure and Physical Activity, Santa Catarina State University, Florianopolis, Brazil.; 6 The University of Queensland, School of Human Movement and Nutrition Sciences, St Lucia, Australia.; 7 Department of Epidemiology, GROW School for Oncology and Developmental Biology, Maastricht University, Maastricht, the Netherlands.; 8 Medical Oncology, St Vincent's Hospital, Melbourne, Australia.; 9 Department of Medicine, The University of Melbourne, Melbourne, Australia.; 10 Bristol Medical School, University of Bristol, Bristol, UK.; 11 NIHR Biomedical Research Centre at University Hospitals Bristol and Weston NHS Foundation Trust and the University of Bristol, Bristol, UK.; 12 Physical Activity Laboratory, Baker Heart and Diabetes Institute, Melbourne, Australia.

## Abstract

The protective effect of physical activity on breast cancer incidence may partially be mediated by inflammation. Systematic searches of Medline, EMBASE, and SPORTDiscus were performed to identify intervention studies, Mendelian randomization studies, and prospective cohort studies that examined the effects of physical activity on circulating inflammatory biomarkers in adult women. Meta-analyses were performed to generate effect estimates. Risk of bias was assessed, and the Grading of Recommendations Assessment, Development, and Evaluation system was used to determine the overall quality of the evidence. Thirty-five intervention studies and one observational study met the criteria for inclusion. Meta-analyses of randomized controlled trials (RCT) indicated that, compared with control groups, exercise interventions reduced levels of C-reactive protein (CRP) [standardized mean difference (SMD) = −0.27, 95% confidence interval (CI) = −0.62 to 0.08), tumor necrosis factor alpha (TNFα, SMD = −0.63, 95% CI = −1.04 to −0.22), interleukin-6 (IL6, SMD = −0.55, 95% CI = −0.97 to −0.13) and leptin (SMD = −0.50, 95% CI = −1.10 to 0.09). Owing to heterogeneity in effect estimates and imprecision, evidence strength was graded as low (CRP, leptin) or moderate (TNFα and IL6). High-quality evidence indicated that exercise did not change adiponectin levels (SMD = 0.01, 95% CI = −0.14 to 0.17). These findings provide support for the biological plausibility of the first part of the physical activity—inflammation—breast cancer pathway.

## Introduction

Women who perform higher levels of physical activity may have a reduced risk of developing breast cancer compared with women with lower levels of physical activity (1–3). Although the mechanisms that underlie this relationship are not clearly understood, inflammation, along with sex steroid hormones and insulin/IGF system signaling, are among the main proposed causal pathways (4, 5).

Inflammation is thought to be a key contributor to cancer growth and progression as it stimulates cell proliferation and influences the tumor microenvironment, enhancing the recruitment, proliferation, and function of protumorigenic auxiliary cells (6, 7). Regular physical activity may reduce inflammation via the production and release of myokines from skeletal muscle tissue (8, 9), reduced levels of body fat, slow age-related weight gain (8, 10), and by reducing production of proinflammatory cytokines by the immune system (8). Evidence from observational studies generally support these hypotheses, demonstrating an inverse relationship between physical activity levels and circulating biomarkers of inflammation in men and women (4, 11). However, the evidence from randomized physical activity intervention trials has been inconsistent. Although some trials report a decrease in inflammatory biomarkers physical activity interventions, others have not identified any changes (4, 11). The reasons for this heterogeneity are multifactorial and do not rule out a causal relationship (e.g., reflecting aspects of the intervention, such as type, intensity, or duration of physical activity; or design aspects such as adherence levels and length of follow-up). Thus, confirming an effect (if any effect), estimating its magnitude, and investigating heterogeneity, may help improve understanding of the mechanisms that underlie relationships of the physical activity with breast cancer risk.

The World Cancer Research Fund (WCRF) International and University of Bristol have developed a causal evidence synthesis framework for conducting systematic reviews of mechanistic pathways for exposure–cancer associations (12). We previously outlined this approach in our protocol paper (5), and applied the method to examine the mechanistic evidence for the sex steroid hormone and insulin/IGF system signaling pathways (13–16). We utilize this framework in the current review to assess the effect of physical activity on inflammatory biomarkers in women. The second part of our systematic review will evaluate the evidence that inflammatory biomarkers are in turn related to breast cancer risk (17). The combined evidence elucidated from both reviews will allow us to evaluate the likelihood that physical activity influences breast cancer risk via an impact on circulating inflammatory biomarkers.

## Materials and Methods

This review has been conducted in accordance with the Preferred Reporting Items for Systematic Reviews and Meta-Analyses (PRISMA) statement (18) and registered with PROSPERO (CRD4202165689), and a detailed protocol paper has been published (5). In brief, systematic searches of Medline (Ovid), EMBASE (Ovid), and SPORTDiscus were performed on February 23, 2021 (Supplementary Methods and Material; Table 1). Peer-reviewed randomized controlled trials (RCT), prospective cohort studies and Mendelian randomization studies were eligible for inclusion if they examined the effect of physical activity on circulating inflammatory biomarkers in postmenarche women. Inflammatory biomarker outcomes were identified via a novel text mining tool, TeMMPo (Text Mining for Mechanism Prioritization; ref. 19), in consultation with experts in the field (5). Biomarkers included: C-reactive protein (CRP); tumor necrosis factor alpha (TNFα); IL1, IL6, IL8, IL10, IL13, and IL1β, IFNγ chemokine ligand 2; adiponectin; and leptin. Risk of bias was assessed using the Cochrane Collaboration Tool for RCTs (20); ROBINS-I (Risk of Bias in Nonrandomized Studies of Interventions) for nonrandomized interventions; and ROBINS-E (Risk of Bias in Nonrandomized Studies of Exposures) for observational studies (21, 22). Noting there are no established tools to assess risk of bias in Mendelian randomization studies, we used the STROBE-MR to appraise reporting quality of included Mendelian randomization studies (23). The overall quality of evidence as well as the strength of findings for each physical activity—inflammatory biomarker relationship was appraised using the Grading of Recommendations Assessment, Development, and Evaluation (GRADE) system (24). For all extracted outcomes, data were summarized and presented descriptively. Where study design, exposures, outcomes, and analyses were defined consistently in at least three separate studies, random-effects meta-analysis was used to generate a pooled standardized mean difference (SMD) with 95% confidence interval (CI). When greater than moderate heterogeneity (*I*^2^ > 30%) was identified, subgroup analysis or meta-regression were performed if the number of available studies (≥10 studies) permitted it. The subgroups were: menopausal status and exercise type. Variables considered in meta-regression included participant age, body mass index (BMI), and physical activity duration and intensity. All statistical analyses were performed using Stata version 16 (Stata Corporation).

**Table 1.** GRADE appraisal for physical activity–inflammatory biomarkers pathways.

Outcome	Meta-analysis study *n* (participant *n*)	Meta-analysis effect estimate SMD (95% CI)	GRADE judgment
CRP	12 (1, 210)	−0.27 (−0.62 to 0.08)	Low^a^^,^^b^
**Cytokines**			
TNFα	8 (564)	−0.63 (−1.04 to −0.22)	Moderate^a^
IL1B	NA	NA	Very low
IL6	11 (895)	−0.55 (−0.97 to −0.13)	Moderate^a^
IL8	NA	NA	Very low
IL10	NA	NA	Very low
**Adipokines**			
Adiponectin	5 (645)	0.01 (−0.14 to 0.17)	High
Leptin	4 (586)	−0.50 (−1.10 to 0.09)	Low^a^^,^^b^

Abbreviations: CI, confidence interval; CRP, c-reactive protein; IL = interleukin; SMD, standardized mean difference (Hedges G); TNFα, tumor necrosis factor-alpha.

^a^Graded down due to high heterogeneity.

^b^Graded down due to imprecision.

## Results

### Search results

**Figure 1.**
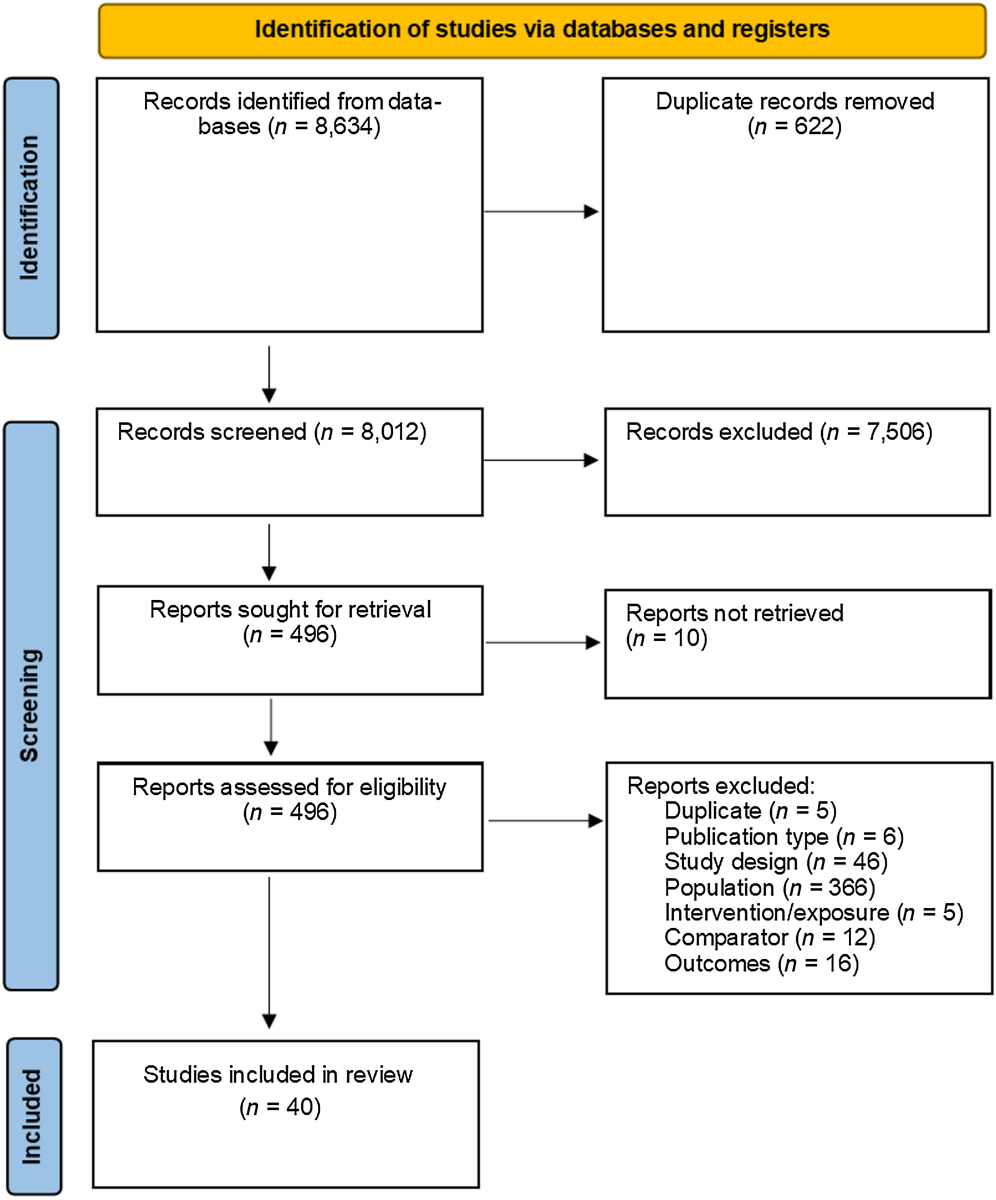
PRISMA flow diagram. This figure incorporates literature search, screening, and study selection. Reasons for full-text inclusion included duplicate study, publication type (e.g., review), study design (e.g., cross-sectional design), population (e.g., male only), intervention/exposure (e.g., physical activity and diet), comparator (e.g., diet or medication), and outcomes (e.g., no inflammation measures).

Results of the systematic search and screening are presented in Fig. 1. From 8,634 records identified, 40 full-text articles met the criteria for inclusion. The most common reasons for full-text exclusion included study design (e.g., cross-sectional analyses), wrong population (e.g., males only or male and female data not stratified), inappropriate comparator (e.g., diet condition), or wrong outcomes (e.g., no inflammatory biomarkers). These included 23 parallel group RCTs (26 publications; refs. 25–50), three randomized cross-over trials (51–53), 9 nonrandomized interventions (10 publications; refs. 54–63), and one observational study (64).

### Study characteristics

The study characteristics tables are presented in Supplementary Methods and Material (Supplementary Tables S2A–S2D) and summarized in Table 1. Studies included either premenopausal (RCTs = 7, cross-over trials = 1, nonrandomized intervention = 7, observation study = 1), perimenopausal (nonrandomized intervention = 1), or postmenopausal (RCTs = 16, cross-over trials = 2, nonrandomized intervention = 3) women. The sample size ranged from 16 to 389 women in RCTs; 12 to 32 in cross-over studies, and 12 to 30 in nonrandomized interventions, with an observational study involving 58 participants. The interventions prescribed aerobic exercise (RCTs = 15, cross-over trials = 12, nonrandomized intervention = 6), resistance exercise (RCTs = 7, cross-over trials = 1, nonrandomized intervention = 2), combined aerobic and resistance exercise (RCTs = 2), plyometric exercise (nonrandomized intervention = 1), and core exercise (RCT = 1). Exercise interventions included acute exercise (i.e., a single exercise session, cross-over trials = 2, nonrandomized interventions = 8) or ongoing exercise (RCT = 21, cross-over trials = 1, nonrandomized interventions = 1). Of the ongoing interventions, the median intervention duration was 16 weeks, and the range was 8 to 52 weeks. The exposure in the observational study was accelerometer-measured physical activity energy expenditure. The comparison condition included an inactive/usual activity control (RCT = 20, cross-over trials = 1, nonrandomized intervention = 2), an exercise intervention of a different type or structure (RCT = 4, cross-over trials = 1), lower exercise dose or intensity (RCT = 2, cross-over trials = 1) different participant age group or menopause status (nonrandomized intervention = 3), menstrual cycle phase (nonrandomized interventions = 2), or anthropometry (nonrandomized interventions = 1). Outcomes included circulating CRP (RCT = 18, cross-over trials = 1, nonrandomized interventions = 3), TNFα (RCT = 12, nonrandomized intervention = 7, observational study = 1), IL1β (RCTs = 1, nonrandomized intervention = 2, observational study = 1), IL6 (RCTs = 15, cross-over trials = 1, nonrandomized interventions = 7, observational study = 1), IL8 (nonrandomized intervention = 1, observational study = 1), IL10 (RCTs = 2, nonrandomized intervention = 1), adiponectin (RCTs = 8, nonrandomized intervention = 1) and leptin (RCTs = 6).

### Risk of bias

The risk-of-bias scores are presented in Supplementary Methods and Material (Supplementary Tables S3A–S3C). All RCTs and cross-over trials were judged to have a high risk of performance bias owing to the difficulty in blinding participants to their exercise intervention. A further nine RCTs were judged to have high attrition bias owing to poor intervention compliance and/or completion rates (i.e., <90% adherence or completion; refs. 27, 29, 34, 36, 42, 46–48, 50). In addition, one RCT and two cross-over trials were judged to have a high risk of bias as they did not clearly present the assay type, sensitivity, or reliability parameters (26, 52, 53). One RCT was also judged to have a high risk of bias as it did not clearly present control group outcomes (26). All nonrandomized interventions had at least moderate risk of bias owing to the potential of confounding explaining any observed physical activity—inflammation relationship. One nonrandomized intervention was judged to have a serious risk of bias as it did not present information about participant body composition or anthropometry (55). One RCT, two cross-over studies, and one nonrandomized intervention had an increased risk of bias as they did not provide clear information on assay sensitivity (26, 52, 53, 59). The prospective cohort study was judged to have a moderate risk of bias owing to the likely presence of confounding (64).

### Effect of physical activity on inflammatory biomarkers

**Figure 2.**
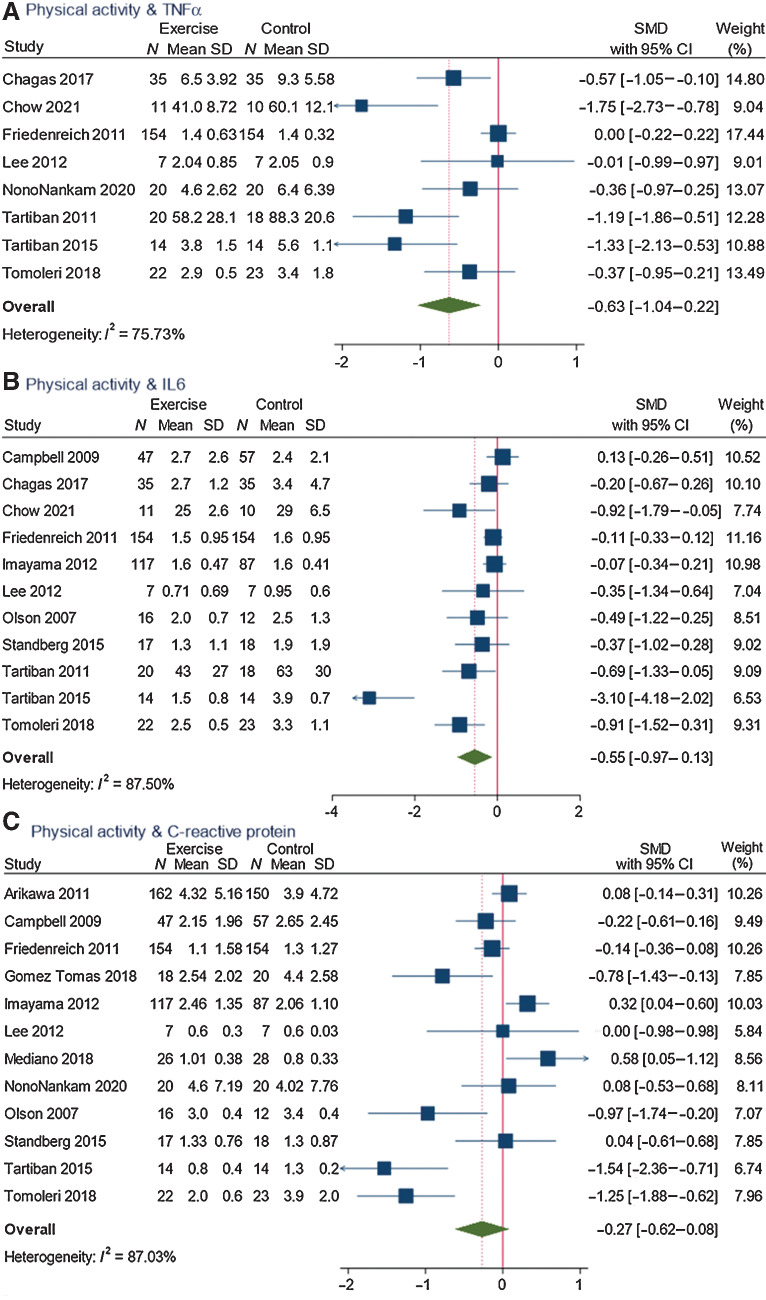
Forest plots for effects of physical activity compared with usual activity control in RCTs. A forest plot for (**A**) TNFα, (**B**) IL6, and (**C**) CRP.

**Figure 3.**
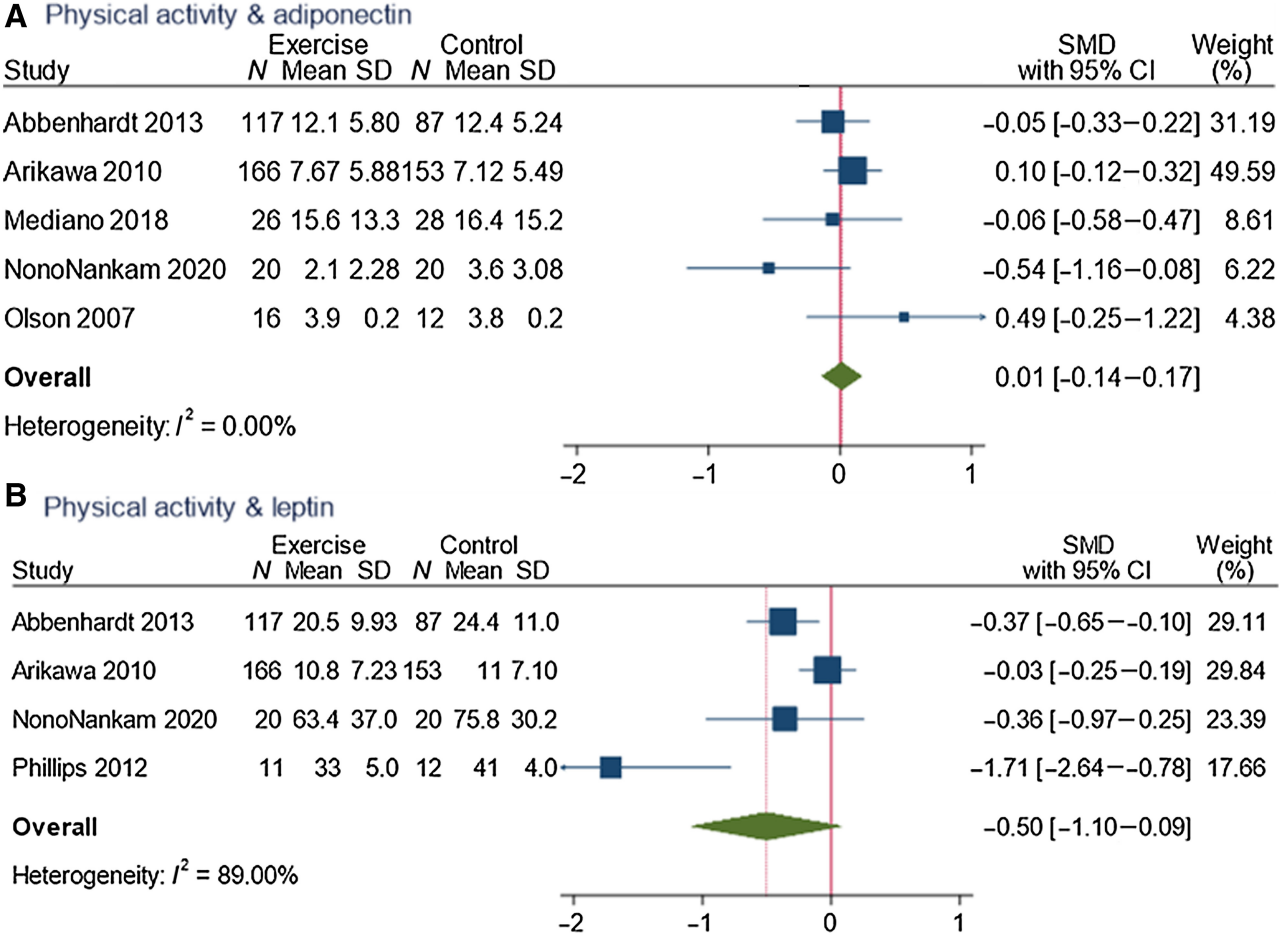
Forest plots for effects of physical activity interventions compared with usual activity in RCTs. A forest plot for (**A**) adiponection and (**B**) leptin.

Meta-analysis was performed for RCTs that compared the effects of ongoing exercise interventions on CRP, TNFα, IL6, adiponectin, and leptin. Meta-analysis results are presented in Fig. 2 (CRP and cytokines) and Fig. 3 (adipokines). Subgroup, meta-regression, and funnel plot results are presented in Supplementary Methods and Materials (Supplementary Figs. S1–S5; Tables 4–5). Results from individual studies that were not included in the meta-analyses are also presented in Supplementary Tables S6A–S6D.

### CRP

Circulating CRP was lower following exercise interventions (12 studies, *n* = 1,210; SMD = −0.27; 95% CI = −0.62 to 0.08; *I*^2^ = 87%) compared with control. However, there was high heterogeneity among studies, a confidence interval that crossed the null, and potential publication bias evident in the funnel plot.

Subgroup analysis could not explain the heterogeneity, as this remained high for each subgroup including pre- (*I*^2^ = 70%) and postmenopausal women (*I*^2^ = 89%), as well as aerobic (*I*^2^ = 89%) and resistance training (*I*^2^ = 64%). Meta-regression was also unable to explain the observed heterogeneity, as there was no clear relationship between the study effect estimate and mean participant age, mean BMI, intervention duration, or intervention intensity (Supplementary Table S4).

Individual studies not included in the meta-analysis mostly supported a decrease in CRP following prescribed exercise relative to a usual activity control (26, 32, 33, 41, 45, 50), although results of one study indicated that these decreases may not be sustained long term (24 months; ref. 33). In one study, there was no clear difference in the effect of a moderate or high dose (i.e., 150 vs. 300 minutes per week) of aerobic exercise; however, this may have been affected by participant adherence, as greater time spent in the target heart rate zone was associated with greater reductions in CRP (32). No difference was identified in the CRP response to exercise according to the menstrual cycle phase (54, 62).

### Cytokines

Meta-analysis of RCTs identified a decrease in TNFα (8 studies, *n* = 564, SMD = −0.63, 95% CI = −1.04 to −0.22, *I*^2^ = 76%) and IL6 (11 studies, *n* = 895, SMD = −0.55, 95% CI = −0.97 to −0.13, *I*^2^ = 88%), following exercise interventions compared with control. Meta-analysis also identified high heterogeneity and potential publication bias evident in the funnel plots.

For TNF—a, heterogeneity remained high in subgroup analysis for pre- (*I*^2^ = 75%) and postmenopausal women (*I*^2^ = 79%), as well as aerobic (*I*^2^ = 84%) and resistance training (*I*^2^ = 91%). There were too few studies to allow meta-regression to explore heterogeneity for TNF-a. For IL6, heterogeneity remained high in the subgroup analysis of studies that included only postmenopausal women (*I*^2^ = 94%) or prescribed only aerobic training (*I*^2^ = 59%) but not for studies of premenopausal women (*I*^2^ = 0%) or resistance training (*I*^2^ = 0%). Meta-regression indicated that participant BMI at baseline explained some of the heterogeneity in the observed IL6 effect estimates (β (95% CI) = 0.17 (0.03–0.30), as did intervention duration [β (95%CI) = 0.02, 95% CI = 0.01–0.03].

In individual studies not included in the meta-analyses, both TNFα and IL6 levels decreased following aerobic and resistance exercise interventions (31, 32, 45, 50, 59, 61). The decrease was not affected by aerobic exercise dose (32); however, for IL6, more time spent exercising at higher intensities was associated with greater decreases (32). In one study, the decrease in IL6 was more evident in participants with a larger compared with a smaller waist size (53). Three nonrandomized interventions showed an increase in IL6 levels following acute exercise (55, 56, 58).

In individual studies, acute exercise preceded an increase in IL1β and IL10 and a decrease in IL8 (55, 59, 63). Ongoing exercise resulted in a decrease of IL1β, no evidence of change in IL8, and an increase in IL10 (29, 42, 45, 47). There were too few studies to facilitate meta-analyses for cytokines IL1, IL1β1β, IL8, IL10, or IL13.

One observational study examined whether physical activity energy expenditure predicted prospective changes in IL1β, IL6, and IL8 (64). No evidence of any relationships was reported for these outcomes.

### Adipokines

No evidence of any changes in adiponectin following exercise interventions compared with control was identified in the meta-analysis (5 studies, *n* = 645, SMD = 0.01, 95% CI = −0.14, 0.17, *I*^2^ = 0%). In contrast, there was evidence of a decrease in leptin following exercise (4 studies, *n* = 586, SMD = −0.50, 95% CI = −1.10, 0.09, *I*^2^ = 89%). However, for leptin, meta-analysis identified high heterogeneity between studies, confidence intervals that crossed the null, and possible publication bias (Supplementary Fig. SF5). There were too few studies to investigate heterogeneity for leptin as an outcome.

In individual studies not included in the meta-analysis, there was little evidence that adiponectin levels changed following acute exercise, but there was evidence that levels increased following ongoing aerobic or core exercise (44, 50, 57). There was little evidence that leptin levels changed following acute exercise but there was evidence that levels decreased following ongoing exercise (44, 58).

## GRADE

GRADE appraisal results are presented in Table 1. The evidence for an effect of physical activity on CRP, TNFα, IL6, adiponectin, and leptin in women was initially graded as high, as findings were based on meta-analyses of RCTs. The GRADE rating for physical activity to TNFα and IL6 was downgraded to moderate owing to high heterogeneity in effect estimates between studies. The ratings for CRP and leptin were downgraded to low owing to high heterogeneity in effect estimates between studies and lower certainty attributable to wide confidence intervals. The evidence for an effect of physical activity on IL1, IL1β1β, IL8, and IL10 was graded as very low, as there were insufficient studies to perform meta-analysis or support any definitive conclusions.

## Discussion

Meta-analysis identified decreases in circulating CRP, TNF-a, IL6, and leptin following exercise interventions compared with control. Effect sizes indicated a small (leptin) or medium (CRP, TNF-a, IL6) sized effect, but the quality of evidence was graded as low (CRP, leptin) or moderate (TNF-a, IL6) owing to high heterogeneity, wide confidence intervals, and potential publication bias. There was high-quality evidence that adiponectin did not change following exercise intervention. Our results support a likely decrease in select inflammatory biomarkers in response to physical activity; however, the level of confidence in this effect is limited.

Several strengths and limitations should be considered when interpreting the findings of this review. A key strength is the robust methodology employed to identify, synthesize, and appraise the evidence for the physical activity–inflammatory biomarker pathway in women. Conclusions are primarily based on meta-analysis results of RCTs, which is also a strength, as RCTs often provide a strong level of evidence. However, as the included RCTs focused more on the efficacy of structured exercise programs and less on physical activity completed in real-world settings, the effectiveness of real-world physical activity on inflammatory biomarkers remains unclear. Although observational and Mendelian randomization studies were eligible for inclusion, only one observational study identified by the systematic search was considered relevant. The greater availability of epidemiologic studies may have provided additional insight into the relationship between physical activity and inflammation in women at a population level. Several individual RCTs were limited by intervention completion and adherence, further complicating the ability to discern true effects. The strict study inclusion criterion, i.e., only including postmenarche women free from disease, is a review strength as it limits potential sources of bias. However, it may have limited the quantity of evidence available as well as the potential generalizability of review findings. As with all systematic reviews, it is possible that some relevant literature was not identified by the search strategy employed. However, following a review of reference lists for included papers and comparison with other reviews, we are confident that the results provide an accurate synthesis of current evidence.

The overall findings of our review are like those that examined the relationship between exercise training on inflammation in animals, older persons, or postmenopausal women (9, 65–68). Although this implies robust findings, we note our review identified smaller effect estimates for outcomes CRP and IL6 compared with the review by Khalafi and colleagues (65). This difference may be due to study inclusion criteria: although our review only included women who were free from disease, Khalafi and colleagues (65) included several studies of women that had cancer or metabolic syndrome at baseline. Including participants with preexisting disease may imply higher levels of inflammation at baseline and greater potential for exercise-induced changes.

Subgroup analysis and meta-regression were unable to provide clear insight into the reasons for the high heterogeneity observed. For most inflammatory biomarkers, heterogeneity remained high within trials that examined only pre- or postmenopausal women or specific types of exercise. Meta-regression did suggest that mean participant BMI at baseline was related to the size of an exercise—IL6 effect, which may suggest a mediating role of body composition. This finding was supported by those of individual RCTs included in our review, where fat loss was identified as a mediator of some exercise—inflammation effects (28, 31). Further, there was no change evident in adiponectin, which is produced by adipocytes, and only low-quality evidence of a small change in leptin, also produced by adipocytes.

Several recommendations can be made to improve the evidence quality for physical activity and inflammatory biomarkers in women. Given the low to moderate evidence strength, additional RCTs are still needed to clarify the effects of exercise on inflammation and to better understand reasons for variability within participant responses. Causal mediation analysis can be used to provide greater insight into the direct effects and indirect effects (e.g., via anthropometric change) of physical activity on inflammatory markers. Interaction analysis may also provide new information on who is likely to experience inflammation change in response to physical activity. Further, as meta-analysis was only possible for a select number of potentially relevant biomarkers identified via TeMMPo, there is a need for physical activity studies to expand the scope of inflammatory outcomes examined. Myokines, which are released by skeletal muscle in response to exercise (9), are proposed to have an anti-inflammatory effect and may contribute to the regulation of tumor growth (69), were not mechanisms prioritized by TeMMPo. As TeMMPo prioritizes mechanisms based on the quantity of evidence, this suggests the evidence base for a physical activity–myokines–breast cancer pathway still requires development. Evidence that physical activity protects against breast cancer is predominantly based on findings from observational studies that use measures of real-world physical activity (2). Despite this, most evidence for this mechanistic pathway came from trials that feature carefully prescribed exercise interventions. Information on the benefit of strategies like breaking up sitting time or accruing physical activity outside of structured exercise is needed to inform public health guidelines.

Physical activity is unlikely to affect breast cancer risk via a single pathway. This review is part of a series of reviews examining the evidence for potential mechanistic pathways that may explain the physical activity–breast cancer relationship (5). Our previous reviews have identified strong evidence for a physical activity–sex steroid hormones–breast cancer pathway. They also identified strong evidence for an effect of physical activity on insulin but not for insulin on breast cancer risk. Compared with sex steroid hormones and insulin signaling (14, 15), the effect estimates for physical activity and inflammatory biomarkers were generally higher, but the quality of evidence was lower and estimates less precise. Despite limitations in evidence quality, overall, findings mostly suggest a decrease in inflammation after physical activity. Part 2 of this review will examine the evidence for these inflammatory biomarkers as contributors to breast cancer risk (17).

## Supplementary Material

Figure S1A Supplementary Figure 1A presents forest plots for physical activity and CRP, by menopausal status

Figure S1B Supplementary Figure 1B presents forest plots for physical activity and CRP, by exercise type

Figure S2A Supplementary Figure 2A present forest plots for physical activity and TNF-alpha, by menopausal status

Figure S2B Supplementary Figure 2B presents forest plots for physical activity and TNF-alpha, by exercise type

Figure S3A Supplementary Figure 3A presents forest plots for physical activity and IL-6, by menopausal status

Figure S3B Supplementary Figure 3B presents forest plots for physical activity and IL-6, by exercise type

Figure S4 Supplementary Figure 4 presents funnel plots for physical activity to inflammatory biomarkers meta-analyses

Table S1 Supplementary Table 1 presents the search terminology used in the systematic review

Table S2A Supplementary Table 2A presents the study characteristics for parallel group RCTs

Table S2B Supplementary Table 2B presents the study characteristics of randomised cross-over trials

Table S2C Supplementary Table 2C presents the study characteristics for non-randomised interventions

Table S2D Supplementary Table 2D presents the study characteristics of the prospective cohort study

Table S3A Supplementary Table 3A presents the risk of bias for randomised controlled and randomised cross-over trials, using the Cochrane Collaboration Tool

Table S3B Supplementary Table 3B presents the risk of bias for non-randomised interventions, using the ROBINS-I

Table S3C Supplementary Table 3C present the risk of bias for the prospective cohort study, using the ROBINS-E

Table S4 Supplementary Table 4 presents the outcomes of the meta-regression analysis conducted for CRP

Table S5 Supplementary Table 5 presents the outcomes of the meta-regression analysis conducted for IL-6

Table S6A Supplementary Table 6A presents findings of individual parallel group RCTs

Table S6B Supplementary Table 6B presents findings of individual randomised cross-over studies

Table S6C Supplementary Table 6C presents findings of individual non-randomised interventions

Table S6D Supplementary Table 6D presents findings of the prospective cohort study
