# Supplementary material for: Linking Physical Activity to Breast Cancer Risk via Inflammation, Part 1: The Effect of Physical Activity on Inflammation
Source: Cancer Epidemiol Biomarkers Prev. 2023 Mar 3;32(5):588–96. doi: 10.1158/1055-9965.EPI-22-0928 (PMC10150243; doi:10.1158/1055-9965.EPI-22-0928)
Supplement: Figure S2B — Supplementary Figure 2B presents forest plots for physical activity and TNF-alpha, by exercise type [file epi-22-0928_figure_s2b_suppsf2b.docx]

Supplementary Figure 2B: TNF-α subgroup analysis (exercise type)
