# Supplementary material for: Linking Physical Activity to Breast Cancer Risk via Inflammation, Part 1: The Effect of Physical Activity on Inflammation
Source: Cancer Epidemiol Biomarkers Prev. 2023 Mar 3;32(5):588–96. doi: 10.1158/1055-9965.EPI-22-0928 (PMC10150243; doi:10.1158/1055-9965.EPI-22-0928)
Supplement: Figure S3B — Supplementary Figure 3B presents forest plots for physical activity and IL-6, by exercise type [file epi-22-0928_figure_s3b_suppsf3b.docx]

Supplementary Figure 3B: IL-6 subgroup analysis (exercise type)
