# Supplementary material for: Linking Physical Activity to Breast Cancer Risk via Inflammation, Part 1: The Effect of Physical Activity on Inflammation
Source: Cancer Epidemiol Biomarkers Prev. 2023 Mar 3;32(5):588–96. doi: 10.1158/1055-9965.EPI-22-0928 (PMC10150243; doi:10.1158/1055-9965.EPI-22-0928)
Supplement: Table S1 — Supplementary Table 1 presents the search terminology used in the systematic review [file epi-22-0928_table_s1_suppst1.docx]

Supplementary Table 1: Search terminology

| **Physical activity/ Exercise** | **Inflammation** |
| --- | --- |
| Exercise, physical activity, plyometric exercise, circuit-based exercise, exercise warm-up, exercise cool-down, exercise, exercise movement techniques, youth sports, sports, water sports, racquet sports, snow sports, swimming, baseball, weightlifting, dancing, running, jogging, athletic performance, walking, stair climbing, gymnastics, muscle stretching exercises, high-intensity interval training, bicycling, exercise therapy, physical conditioning, human, yoga | Inflammation mediators, inflammation, anti-inflammatory agents, immune system, cytokines, cytokine*, immun*, acute-phase proteins, acute-phase reaction, adipokines, tumour necrosis factor-alpha cytokine*, interleukin*, lymphocyte*, leptin, leukocyte*, anti-inflammatory cyclooxygenase inhibitor* c-reactive protein, interleukins, interferon-gamma, prostaglandins, chemokines |
