# Supplementary material for: Linking Physical Activity to Breast Cancer Risk via Inflammation, Part 1: The Effect of Physical Activity on Inflammation
Source: Cancer Epidemiol Biomarkers Prev. 2023 Mar 3;32(5):588–96. doi: 10.1158/1055-9965.EPI-22-0928 (PMC10150243; doi:10.1158/1055-9965.EPI-22-0928)
Supplement: Table S2A — Supplementary Table 2A presents the study characteristics for parallel group RCTs [file epi-22-0928_table_s2a_suppst2a.docx]

Supplementary Table 2A. Study characteristics for parallel group randomised control trials.

| **Author, year, country** | **Participants** | **Intervention** | **Comparison** | **Outcome** |
| --- | --- | --- | --- | --- |
| Alhindawi, 2013, Jordan | Post-menopausal women who were physically inactive, N = 20; Age ~ 49, BMI ~35. | Aerobic exercise performed for 60 minutes at 75% HRR, 3 times per week, for 14 weeks. | Inactive control. | CRP |
| WISER Study  Arikawa, 2010, USA | Women In Steady Exercise Research (WISER) study; pre-menopausal, eumenorrheic, physically inactive (≤ 2 weekly sessions of moderate intensity exercise), women aged 18-30 yrs.), N = 391 (exercise = 212, control = 179), Age = 25.3(3.4), BMI = 24.7(4.7) | Aerobic exercise performed for 30 minutes at 70 – 85% HR max, 5 days per week for 4 menstrual cycles/ 14 – 18 weeks. | Inactive control. | Leptin  Adiponectin  CRP |
| Campbell, 2009, USA | Post-menopausal women who were physically inactive. N = 115 (exercise = 53, control = 62), Age ~ 61, BMI ~ 30. | Aerobic exercise performed for 45 minutes at 60-75% HR max, 5 days per week, for 12 months. Sessions were a mix of supervised and unsupervised. | Inactive/ stretching control. | CRP  IL-6 |
| Chagas, 2017, Brazil | Post-menopausal women who were physically inactive, N = 82 (exercise = 44, control = 38), Age ~ 60, BMI ~ 31. | Aerobic training performed for 75 minutes at 50-60% VO2 max, 3 days per week, for 20 weeks. | Inactive control. | TNFα  IL-6  Il-10  IL-6/IL-10  IL-10/ TNFα |
| Chow, 2021, China | Pre-menopausal women who were inactive and had obesity (>30% BF%), N = 36 (D= 12, A = 12, Control = 12), Age ~ 19, BMI ~ 25 | Aerobic exercise that was either descending or ascending stairs. Participants performed 3 sessions per week for 12 weeks. The load gradually increased throughout the sessions | Comparison between ascenders, descenders, and inactive control. | TNFα  IL-6 |
| The ALPHA Trial  Friedenreich, 2012, Canada | Alberta Physical Activity and Breast Cancer Prevention Trial (ALPHA), Post-menopausal, sedentary women; n = 320(160 exercise, 159 control), Age = 60.9(5.6), BMI = 29.2(4.4) | Aerobic exercise performed for 45 mins at 70-80% HRR max, 5 days/wk. (3 supervised), for 12 months | Inactive control/ Newsletter and future fitness pass. | CRP  IL-6  TNFα |
| The BETA Trial  Friedenreich, 2016, 2019, Canada | The Breast Cancer and Exercise Trial in Alberta (BETA), Post-menopausal, physically inactive women; N = 382 (193 high volume exercise, 189 moderate volume exercise), Age = 59.5(5.0), BMI = 29.3(4.4) | Moderate: Aerobic exercise performed for 30 mins at 60-80% HRR max, 5 day/wk., for 12 months  High: Aerobic exercise performed for 60 mins at 60-80% HRR max, 5 day/wk., for 12 months | Moderate volume exercise was compared to high volume exercise. | CRP  IL-6  TNFα |
| Gomez-Tomas, 2018, Spain | Post-menopausal women, N = 38 (exercise = 18, control = 20), Age ~71, BMI ~29-30. | Resistance training performed for 50 minutes, 3 days per week, for four months. Intensity monitored according to RPE and periodised throughout. | Inactive control. | CRP |
| Henagan, 2011, USA | Post-menopausal women, N = 23 (exercise = 12, control = 11), Age ~66, BMI ~33. | Resistance training performed 3 days per week for 12 weeks. | Inactive control with health education and craft classes. | TNFα |
| Henriquez 2017, Chile | Post-menopausal women, N = 42 (aerobic = 21, light resistance = 21), Age (range) = 45-60, BMI~31 | Aerobic or light resistance training performed for 40 minutes, three times per week, for 6 months. Aerobic exercise performed at 60-65% of predicted VO2max. Light resistance performed on upper and lower body muscle groups to exhaustion. | Aerobic compared to light resistance. | CRP  IL-6 |
| Lee, 2012, Korea | Pre-menopausal women who were inactive and overweight or obese (BMI > 25), n = 22 (low intensity = 8, high intensity = 7, control = 7), Age ~ 40, BMI ~ 27 | Aerobic exercise performed f3 - 5 times per week for 14 weeks. Exercise intensity was either 50% or 70% VO2max. Exercise dose based on energy output. | Comparison of exercise intensity and to inactive control. | CRP  IL-6  TNFα |
| NEW Study  Abbenhardt, 2013, Imayama, 2012, Mason, 2013, USA | Nutrition and Exercise for Women (NEW) Study. Post-menopausal women who were overweight and inactive, N = 204 (exercise = 117, control = 87, NR = 235), Age ~ 57-58, BMI ~31 | Aerobic exercise performed for 45 minutes, five days per week for 12 months. 3 sessions per week were supervised. The intensity increased to 70-85% of HRmax. | Inactive control. | CRP  IL-6  Leptin  Adiponectin |
| Mediano, 2013, Brazil | Pre-menopausal women who were physically inactive, N = 54 (exercise = 26, control = 28), Age ~ 37/39, BMI ~ 26 | Aerobic home-based exercise performed for 40 minutes, 3 days per week for 12 months. Intensity unclear. | Inactive control. | CRP  Adiponectin |
| Mogharnasi, 2019, Iran | Pre-menopausal women who were overweight or obese. N = 34 (endurance = 12, resistance = 12, control = 10) Age ~ 22, BMI ~ 30 | Aerobic or resistance exercise groups that trained 4 days per week for 8 weeks. Endurance exercise included 20 – 34 minutes of running at 65 – 80% HR max. Resistance exercise targeted major muscle groups and was performed for 20 – 35 minutes per session. | Comparison of endurance, resistance and inactive control. | CRP |
| NonoNankam, 2020, South Africapost | Pre-menopausal women who were black South Africans with obesity, N= 35 (exercise = 20, control = 15), Age (range): 20–35 years old, BMI (range): 30–40 kg m^2^. | Combined aerobic and resistance training performed for 40-60 minutes, 4 days per week, for 12 weeks. Aerobic exercise was performed at 75-80% of HR max. Resistance exercise was mostly body weight training targeting major muscle groups. | Inactive control. | CRP  TNFα  IL-8  Adiponectin  Leptin |
| Olson 2007, USA | Pre-menopausal women who were overweight (BMI > 25), N = 30 (exercise = 15, control = 15), Age (range) = 24 - 44 | Resistance training performed at least twice per week for 12 months. Each session included a warmup and upper and lower body resistance exercises with 3 sets of 8-10 reps | Inactive control. | CRP  IL-6  Adiponectin |
| Ozcan, 2015, Turkey | Pre-menopausal women, N= 40. Age 35 ± 5. | Aerobic or core exercise performed for one hour four days per week for 16 weeks. Aerobic exercise was performed at an intensity of 60 – 70% of HR max. Core exercises prescribed in sets and reps. | Comparison of aerobic exercise to core exercise. | Adiponectin  Leptin |
| Phillips, 2012 | Post-menopausal women who were physically inactive and with obesity, N = 23 (exercise = 11, control = 12). Age = 66(2), BMI = 33. | Resistance training performed three times per week for 12 weeks. 10 exercises targeted major muscle groups. | Inactive control/ ‘social interaction’ intervention | CRP  TNFα  Adiponectin  Leptin  IL-6  IL-10 |
| Strandberg, 2015 | Post-menopausal women who were recreationally active, N=35 (exercise = 17, control = 18, not relevant to this review = 20). Age ~ 68, BMI ~ 24. | Resistance training performed 2 times per week for 24 weeks. Exercises targeted major muscle groups and 8-12 reps per set were performed at a final intensity of 75-85% of 1RM. | Inactive control. | CRP  IL-6 |
| Tartiban, 2011, Iran | Post-menopausal women who were physically inactive at baseline, N = 38 (exercise = 20, control = 18, not relevant to this review = 41), Age ~ 60, BMI ~ 25 (exercise)/ 29 (control) | Aerobic exercise. Walking or jogging for 25-45 minutes at 45 – 65% HR max, 3 to 6 days/ week for 24 weeks. Exercise quantity and intensity increased as the study progressed. | Inactive control. | TNFα  IL-6  PGE_2_ |
| Tartiban, 2015, Iran | Post-menopausal women who were physically inactive at baseline, N = 34 (exercise = 17, control = 17). Age ~ 57, BMI ~ 25. | Aerobic exercise performed at 45-65% HR max for 25 – 45 minutes, 3-6 days per week, for 16 weeks. | Inactive control. | IL-1β  IL-6  TNF-α  CRP |
| Tomeleri, 2020, Brazil | Post-menopausal women, N = 44 (single joint = 14, multi-joint = 15, control = 15), age ~70, BMI ~27. | Resistance training performed three times per week for 12 weeks. Each session included 3 reps of 10-15 reps for 8 exercises. | Resistance vs control. Single joint to multi joint vs multi joint to single joint resistance. | TNF-α  CRP  IL-6 |
| SHAPE Study  Van Gemert, 2015, Netherlands | Sex Hormones and Physical Exercise (SHAPE) study, post-menopausal women, N = 189 (exercise = 96, control = 93), Age = 58.7(4.4), BMI = 26.9(3.3) | Combined aerobic and strength training. Supervised 60-minute group exercise 2 days/ week. Additional individual exercise 30 mins, one day/ week. 16-week intervention. | Inactive control. | CRP  IL-6  Adiponectin  Leptin |
