# Supplementary material for: Linking Physical Activity to Breast Cancer Risk via Inflammation, Part 1: The Effect of Physical Activity on Inflammation
Source: Cancer Epidemiol Biomarkers Prev. 2023 Mar 3;32(5):588–96. doi: 10.1158/1055-9965.EPI-22-0928 (PMC10150243; doi:10.1158/1055-9965.EPI-22-0928)
Supplement: Table S2B — Supplementary Table 2B presents the study characteristics of randomised cross-over trials [file epi-22-0928_table_s2b_supps2tb.docx]

Supplementary Table 2B. Study characteristics of randomised cross-over trials

| **Author, year, country** | **Participants** | **Intervention** | **Comparison** | **Outcome** |
| --- | --- | --- | --- | --- |
| Davis, 2008, UK | Post-menopausal women, N = 12, Age ~ 58, BMI ~ 26 | Aerobic exercise. A single 30-minute treadmill walk at either 50% or 70% HR max. | Comparison of walking intensity. | CRP |
| Lustosa, 2013, Brazil | Post-menopausal women, N= 32, aged >65 years (72 ± 4), prefrail. BMI 29.2 ± 4.2. | Resistance exercise featuring 3 1-hour sessions/week for 10 weeks. Exercises targeted the lower limbs and included: a hip exercise; knee extensors and flexors. | Inactive control. | IL-6 |
| Miles, 2016, USA | Pre-menopausal women who were either normal weight (BMI = 18.5 – 24.9) or had overweight/ obesity (BMI = 25 – 35), N = 23 (normal weight = 12, obese = 11), Age ~ 29. | Aerobic exercise that included either uphill or downhill walking performed for 45 minutes at 60% VO2max. Uphill was performed with a +10% incline, downhill with a -10% incline. | Comparison of low to high waist circumference and uphill compared to downhill walking. | IL-6 |
