# Supplementary material for: Linking Physical Activity to Breast Cancer Risk via Inflammation, Part 1: The Effect of Physical Activity on Inflammation
Source: Cancer Epidemiol Biomarkers Prev. 2023 Mar 3;32(5):588–96. doi: 10.1158/1055-9965.EPI-22-0928 (PMC10150243; doi:10.1158/1055-9965.EPI-22-0928)
Supplement: Table S2C — Supplementary Table 2C presents the study characteristics for non-randomised interventions [file epi-22-0928_table_s2c_suppst2c.docx]

Supplementary Table 2C. Study characteristics of non-randomised interventions

| **Author, year, country** | **Participants** | **Intervention** | **Comparison** | **Outcomes** |
| --- | --- | --- | --- | --- |
| Barba Moreno, 2020, Spain | Pre-menopausal, eumenorrheic women with some endurance training. N = 15, Age ~ 35, BM ~ 58kg, VO2 max ~ 50ml/min/kg. | Aerobic exercise. A single 40-minute session of treadmill running performed at 75% of VO2max during three different menstrual cycle phases. | Comparison of early follicular, mid-follicular, and luteal phases. | CRP |
| Giraldo, 2009, Spain | Pre-menopausal women who were physically inactive. N = 30 (moderate exercise = 15, intense exercise = 15). Age (range) = 20-24. | Aerobic exercise. A single 45-minute session of aerobic exercise at either 55% or 70% VO2max. | Comparison of exercise intensity. | IL-1B  IL-6 |
| Gmiat, 2017, Poland | Pre-menopausal women who were physically inactive and aged ~ 23 years or ~ 42 years. N = 14 (‘young’ = 8, ‘middle-aged’ = 42). | Aerobic exercise/ high intensity circuit training. A seven-minute circuit that included exercises performed for 30 seconds with 10 seconds transition. The circuit was repeated three times. | Comparison of ‘young’ to ‘middle-aged’ women. | TNF-α  IL-6  IL-10 |
| Jamurtus, 2013, Greece | Pre-menopausal women, N = 12, Age ~ 29, BMI ~ 23, VO2 max ~ 36. | Aerobic exercise. Single session of treadmill exercise performed at 60% of VO2max for 45 minutes. | Inactive control/ resting condition | TNF-α  IL-6 |
| Kurgan, 2020, Canada | Pre-menopausal adolescents who were either normal weight (BMI ~ 21) or had obesity (BMI ~33), N = 20 (normal weight = 10, obese = 10), age range = 13 – 17. | Plyometric exercise performed in a single session that included 120 jumps organised into five circuit training stations. | Normal weight compared to obese | TNF-α  IL-6 |
| Phillips, 2008, 2010, USA | Post-menopausal women who were physically inactive, N = 16 (exercise = 9, control = 7, not relevant to this review = 19), Age = 72(6), BMI ~ 26. | Resistance training. A single session of resistance training that featured 10 resistance exercises targeting major muscle groups performed at 80% of 1RM. | Inactive control. | IL-1β  IL-6  TNF-α |
| Riesco, 2013 | Pre (N= 18) and post-menopausal women (N= 17). Sedentary, overweight BMI: 29 – 35kg/m^2^. Pre-menopausal women aged 49 ± 3 yrs. Post-menopausal women 53 ± 2yrs. Eccentric resistance training pe | Aerobic exercise. Three non-consecutive sessions of 45 minutes of walking per week at 60% of heart rate reserve. 16-week program. | Comparison of pre- and post-menopausal women | IL-6  Adiponectin  TNF-α  CRP |
| Romero-Parra, 2020 | Pre-menopausal, eumenorrheic women, N = 19, age = 28.6 ± 5.9 years of age, 163.4 ± 6.1 cm height, 59.6 ± 5.8 kg body mass, 14.8 ± 5.1 kg | Eccentric resistance training in the early follicular, late follicular, and mid-luteal phase. Exercise included 10 sets of 10 reps of plate-loaded barbell parallel back squats, at 60% of their 1RM with 2 mins of rest between sets. | Comparison by menstrual cycle phase | CRP  IL-6  TNF-α |
| Serviente, 2016 | Peri- (N=7) or late post-menopausal (N= 8) women. | Aerobic exercise. Participants walked on a treadmill for 30 min at the 60 – 64% of VO2max. The session began and ended with a 5-min warm up and cool down. | Comparison of peri- and post- menopausal women. | IL-8  TNF-α |
