# Supplementary material for: Linking Physical Activity to Breast Cancer Risk via Inflammation, Part 1: The Effect of Physical Activity on Inflammation
Source: Cancer Epidemiol Biomarkers Prev. 2023 Mar 3;32(5):588–96. doi: 10.1158/1055-9965.EPI-22-0928 (PMC10150243; doi:10.1158/1055-9965.EPI-22-0928)
Supplement: Table S2D — Supplementary Table 2D presents the study characteristics of the prospective cohort study [file epi-22-0928_table_s2d_suppst2d.docx]

Supplementary Table 2D. Study characteristics of prospective cohort study

| **Author, year, country** | **Participants** | **Physical activity assessment** | **Comparison** | **Outcomes** | **Confounding variables** |
| --- | --- | --- | --- | --- | --- |
| MONET Study, Razmjou, 2016 | Pre-menopausal women who were eumenorrheic, N= 58. Age range at follow-up= 47 and 55 (49.6 ± 1,7). BMI range = 20.0-29.9kg/m^2^ | Physical activity energy expenditure measured using an Actical accelerometer. | Level of PA (although unclear). | IL-1β,  IL-6,  IL-8,  TNF-α | Fat mass, fat free mass, waist circumference,m trunk fat, peripheral fat, VO2max, physical activity energy expenditure, ApoB, IL-8., ORM, sTNFR2. |
