# Supplementary material for: Linking Physical Activity to Breast Cancer Risk via Inflammation, Part 1: The Effect of Physical Activity on Inflammation
Source: Cancer Epidemiol Biomarkers Prev. 2023 Mar 3;32(5):588–96. doi: 10.1158/1055-9965.EPI-22-0928 (PMC10150243; doi:10.1158/1055-9965.EPI-22-0928)
Supplement: Table S3A — Supplementary Table 3A presents the risk of bias for randomised controlled and randomised cross-over trials, using the Cochrane Collaboration Tool [file epi-22-0928_table_s3a_suppst3a.docx]

Supplementary Table 3A. Risk of bias for randomised control and randomised cross-over trials session assessed using the Cochrane Collaboration Tool

| **Study** | **Risk of bias item** | | | | | | |
| --- | --- | --- | --- | --- | --- | --- | --- |
|  | **1a**  **Selection bias (random sequence generation)** | **1b**  **Selection bias (allotment concealment)** | **2**  **Performance bias** | **3**  **Detection bias** | **4**  **Attrition bias** | **5**  **Reporting bias** | **6**  **Other bias** |
| Alhindawi 2013 | Unclear | Unclear | High | Low | Low | High  Outcomes for control group not clearly reported | High  Assay type, sensivity , and reliability not reported |
| WISER Study  Arikawa, 2010 | Unclear | Unclear | High | Low | High  Greater than 10% attrition | Low | Low |
| Campbell, 2009 | Unclear | Unclear | High | Low | Low | Low | Low |
| Chagas, 2017, | Unclear | Unclear | High | Low | High  Intervention adherence was 77% | Low | Low |
| Chow, 2021, | Unclear | Unclear | High | Low | Low | Low | Low |
| Davis, 2008 | Unclear | Unclear | High | Low | Low | Low | Low |
| The ALPHA Trial  Friedenreich, 2012 | Low | Low | High | Low | Low | Low | Low |
| The BETA Trial  Friedenreich, 2016, 2019, | Low | Low | High | Low | Low | Low | Low |
| Gomez-Tomas, 2018, | Unclear | Unclear | High | Low | High  Greater than 10% attrition | Low | Low |
| Henagan, 2011 | Unclear | Unclear | High | Low | Unclear | Low | Low |
| Henriquez 2017 | Unclear | Unclear | High | Low | High  Greater than 10% attrition | Low | Low |
| Lee, 2012 | Unclear | Unclear | High | Low | Low | Low | Low |
| Lustosa, 2013 | Unclear | Unclear | High | Low | Low | Low | High  Assay sensitivity not clearly reported |
| NEW Study  Abbenhardt, 2013  Imayama, 2012  Mason, 2013 | Low | Low | High | Low | Low | Low | Low |
| Mediano, 2013 | Unclear | Unclear | High | Low | Low | Low | Low |
| Miles, 2016 | Unclear | Unclear | High | Low | Low | Low | High  Assay sensitivity not clearly reported |
| Mogharnasi, 2019 | Unclear | Unclear | High | Low | Unclear | Low | Low |
| NonoNankam, 2020 | Unclear | Unclear | High | Low | High  Greater than 10% attrition | Low | Low |
| Olson 2007 | Unclear | Unclear | High | Low | Low | Low | Low |
| Ozcan, 2015 | Unclear | Unclear | High | Low | Low | Low | Low |
| Phillips, 2012 | Unclear | Unclear | High | Low | Unclear | Low | Low |
| Strandberg, 2015 | Unclear | Unclear | High | Low | High  Greater than 10% attrition | Low | Low |
| Tartiban, 2011 | Unclear | Unclear | High | Low | Low | Low | Low |
| Tartiban, 2015 | Unclear | Unclear | High | Low | High  > 10% dropouts | Low | Low |
| Tomeleri, 2020 | Unclear | Unclear | High | Low | High  >10% dropouts | Low | Low |
| SHAPE Study  Van Gemert, 2015 | Unclear | Low | High | Low | Low  6 drop out | Low | High  Potential departure from exposure w/ 46 non-compliant |
