# Supplementary material for: Linking Physical Activity to Breast Cancer Risk via Inflammation, Part 1: The Effect of Physical Activity on Inflammation
Source: Cancer Epidemiol Biomarkers Prev. 2023 Mar 3;32(5):588–96. doi: 10.1158/1055-9965.EPI-22-0928 (PMC10150243; doi:10.1158/1055-9965.EPI-22-0928)
Supplement: Table S3B — Supplementary Table 3B presents the risk of bias for non-randomised interventions, using the ROBINS-I [file epi-22-0928_table_s3b_suppst3b.docx]

Supplementary Table 3B: Risk of bias in non-randomised interventions assessed using the ROBINS-I

| **Study** | **Risk of bias item** | | | | | | | **Overall ROB** |
| --- | --- | --- | --- | --- | --- | --- | --- | --- |
|  | **1**  **Confounding*** | **2**  **Participant selection** | **3**  **Intervention**  **classification** | **4**  **Intervention deviation** | **5**  **Missing data** | **6**  **Outcome measurement** | **7**  **Reporting results** |  |
| Barba Moreno, 2020 | Moderate | Low | Low | Low | Low | Low | Low | **Moderate** |
| Giraldo, 2009 | Serious  No Information provided for body composition, medication/ hormone use | Low | Low | Low | Low | Low | Low | **Serious** |
| Gmiat, 2017 | Moderate | Low | Low | Low | Low | Low | Low | **Moderate** |
| Jamurtus, 2013 | Moderate | Low | Low | Low | Low | Low | Low | **Moderate** |
| Kurgan, 2020 | Moderate | Low | Low | Moderate | Low | Low | Low | **Moderate** |
| Phillips, 2008, 2010 | Moderate | Low | Low | Low | Low | Moderate  No information provided for assay sensitivity etc | Low | **Moderate** |
| Riesco, 2013 | Moderate | Low | Low | Low | Low | Low | Low | **Moderate** |
| Romero-Parra, 2020 | Moderate | Low | Low | Low | Low | Low | Low | **Moderate** |
| Serviente, 2016 | Moderate | Low | Low | Low | Low | Low | Low | **Moderate** |
