# Supplementary material for: Linking Physical Activity to Breast Cancer Risk via Inflammation, Part 1: The Effect of Physical Activity on Inflammation
Source: Cancer Epidemiol Biomarkers Prev. 2023 Mar 3;32(5):588–96. doi: 10.1158/1055-9965.EPI-22-0928 (PMC10150243; doi:10.1158/1055-9965.EPI-22-0928)
Supplement: Table S3C — Supplementary Table 3C present the risk of bias for the prospective cohort study, using the ROBINS-E [file epi-22-0928_table_s3c_suppst3c.docx]

Supplementary Table 3C: Risk of bias in observational studies assessed using the ROBINS-E

| **Study** | **Risk of bias item** | | | | | | | **Overall ROB** |
| --- | --- | --- | --- | --- | --- | --- | --- | --- |
|  | **1**  **Confounding** | **2**  **Participant selection** | **3**  **Exposure classification** | **4**  **Exposure departure** | **5**  **Missing data** | **6**  **Outcome assessment** | **7**  **Reporting results** |  |
| Razmjou, 2016 | Moderate  Some potential for confounding but key confounders adjusted for | Low | Low  Physical activity assessed using an accelerometer | Moderate  Potential change in PA levels | Low | Low | Low | **Moderate** |
