# Supplementary material for: Linking Physical Activity to Breast Cancer Risk via Inflammation, Part 1: The Effect of Physical Activity on Inflammation
Source: Cancer Epidemiol Biomarkers Prev. 2023 Mar 3;32(5):588–96. doi: 10.1158/1055-9965.EPI-22-0928 (PMC10150243; doi:10.1158/1055-9965.EPI-22-0928)
Supplement: Table S4 — Supplementary Table 4 presents the outcomes of the meta-regression analysis conducted for CRP [file epi-22-0928_table_s4_suppst4.docx]

Supplementary Table 4: Meta-regression for CRP

| **Moderator** | **B (95% CI)** |
| --- | --- |
| Mean participant age | -0.01 (-0.03, 0.01) |
| Mean participant BMI | 0.03 (-0.10, 0.15) |
| Intervention weeks | 0.01 (-0.01, 0.03) |
| Intervention intensity (aerobic exercise only) | 0.03 (-0.02, 0.07) |
