# Supplementary material for: Linking Physical Activity to Breast Cancer Risk via Inflammation, Part 1: The Effect of Physical Activity on Inflammation
Source: Cancer Epidemiol Biomarkers Prev. 2023 Mar 3;32(5):588–96. doi: 10.1158/1055-9965.EPI-22-0928 (PMC10150243; doi:10.1158/1055-9965.EPI-22-0928)
Supplement: Table S5 — Supplementary Table 5 presents the outcomes of the meta-regression analysis conducted for IL-6 [file epi-22-0928_table_s5_suppst5.docx]

Supplementary Table 5: Meta-regression for IL-6

| **Moderator** | **B (95% CI)** |
| --- | --- |
| Mean participant age | 0.01 (-0.03, 0.04) |
| Mean participant BMI | 0.17 (0.03, 0.30) |
| Intervention weeks | 0.02 (0.01, 0.03) |
| Intervention intensity (aerobic exercise only) | 0.02 (-0.01, 0.05) |
