# Supplementary material for: Linking Physical Activity to Breast Cancer Risk via Inflammation, Part 1: The Effect of Physical Activity on Inflammation
Source: Cancer Epidemiol Biomarkers Prev. 2023 Mar 3;32(5):588–96. doi: 10.1158/1055-9965.EPI-22-0928 (PMC10150243; doi:10.1158/1055-9965.EPI-22-0928)
Supplement: Table S6A — Supplementary Table 6A presents findings of individual parallel group RCTs [file epi-22-0928_table_s6a_suppst6a.docx]

Supplementary Table 6A: Findings of individual parallel group randomised controlled trials

| **Study** | **Finding** |
| --- | --- |
| Alhindawi 2013 | **CRP** decreased following training in the exercise group, but control group numbers not clearly presented. |
| Chagas 2017 | **IL-10** decreased in both the intervention and control group. The decrease was more pronounced in the control group. |
| Friedenreich 2016, 2019 (BETA trial) | **CRP, IL-6** and **TNF-α** decreased following moderate and high doses of aerobic exercise. There was no definitive effect of exercise dose. **CRP** decreases were not sustained over time. |
| Henagan 2011 | **TNF-α increased** in both control and resistance training groups. |
| Henriquez 2017 | **CRP** increased following endurance training and decreased following resistance training. Changes were not significant. |
| Mogharnasi 2019 | Endurance and resistance training preceded large decreases in high sensivitive **CRP** compared to control. |
| Nono Namkam 2020 | There was no change in **IL-8** in response to exercise. |
| Orzcan 2015 | **Adiponectin** increased after aerobic and core exercise. **Leptin** decreased after 8 weeks exercise but returned to baseline after 16 weeks. |
| Phillips 2012 | Resistance training decreased **CRP**and **TNF-α.** It increased **IL-10**. |
| Tartibian 2015 | IL-1B decreased in response to exercise. |
| Van Gemert 2015 (SHAPE Study) | Significant decreases in **CRP** and **Leptin** and non-significant decreases in **IL-6** for exercise compared to control. Increase in **adiponectin**. |
