# Supplementary material for: Linking Physical Activity to Breast Cancer Risk via Inflammation, Part 1: The Effect of Physical Activity on Inflammation
Source: Cancer Epidemiol Biomarkers Prev. 2023 Mar 3;32(5):588–96. doi: 10.1158/1055-9965.EPI-22-0928 (PMC10150243; doi:10.1158/1055-9965.EPI-22-0928)
Supplement: Table S6B — Supplementary Table 6B presents findings of individual randomised cross-over studies [file epi-22-0928_table_s6b_suppst6b.docx]

Supplementary Table 6B: Findings of individual randomised cross-over studies

| **Study** | **Finding** |
| --- | --- |
| Davis 2008 | No change in **CRP** following acute exercise |
| Lustosa 2015 | No change in **IL-6** following exercise |
| Miles 2016 | **IL-6** did not change following acute exercise in ‘low waist circumference’ participants but did decrease in ‘high waist circumference’ participants. |
