# Supplementary material for: Linking Physical Activity to Breast Cancer Risk via Inflammation, Part 1: The Effect of Physical Activity on Inflammation
Source: Cancer Epidemiol Biomarkers Prev. 2023 Mar 3;32(5):588–96. doi: 10.1158/1055-9965.EPI-22-0928 (PMC10150243; doi:10.1158/1055-9965.EPI-22-0928)
Supplement: Table S6C — Supplementary Table 6C presents findings of individual non-randomised interventions [file epi-22-0928_table_s6c_suppst6c.docx]

Supplementary Table 6C: Findings of individual non-randomised controlled trials

| **Study** | **Finding** |
| --- | --- |
| Barba Moreno, 2020, Spain | Although baseline **CRP** levels varied according to menstrual cycle stage, there was no clear change following exercise. |
| Giraldo, 2009, Spain | Following moderate intensity exercise**, IL-1B** and **IL-6** increased. Following intense exercise, IGN-y, **IL-1B**, and **IL-6** increased while IL-4 decreased. |
| Gmiat, 2017, Poland | There were moderate increases in **IL-6** and **IL-10** following exercise in both younger and middle-aged participants. **TNF-α** also increased in younger participants but decreased in older participants following exercise. The effect sizes were moderate for both groups. Only **TNF-α** was different between groups. |
| Jamurtus, 2013, Greece | **Adiponectin** did not change following exercise. |
| Kurgan, 2020, Canada | **IL-6** and **TNF-α** increased following acute exercise in both lean and obese participants before returning to baseline levels after 24 hours. There was no difference in magnitude of response between groups. **Leptin** did not change after exercise in either group. |
| Phillips, 2008, 2010, USA | Resistance training decreased circulating **TNF-α** as well as production of **IL-6**, **IL-1B**, and **TNF-α** . |
| Riesco, 2013 | There were decreases in **IL-6** following exercise in both pre- and post-menopausal women. There were no clear changes in **TNF-α** or **adiponectin** following exercise. |
| Romero-Parra, 2020 | No clear changes in **IL-6**, **TNF-α,** or **CRP** following eccentric resistance exercise and no clear difference in response according to menstrual cycle phase. |
| Serviente, 2016 | **TNF-α** and **IL-8** decreased following acute exercise in peri-menopausal and post-menopausal women. The decreases were significantly greater in peri-menopausal women. |
