# Supplementary material for: Linking Physical Activity to Breast Cancer Risk via Inflammation, Part 1: The Effect of Physical Activity on Inflammation
Source: Cancer Epidemiol Biomarkers Prev. 2023 Mar 3;32(5):588–96. doi: 10.1158/1055-9965.EPI-22-0928 (PMC10150243; doi:10.1158/1055-9965.EPI-22-0928)
Supplement: Table S6D — Supplementary Table 6D presents findings of the prospective cohort study [file epi-22-0928_table_s6d_suppst6d.docx]

Supplementary Table 6D: Findings of the prospective cohort study

| **Study** | **Finding** |
| --- | --- |
| Razmjou, 2016 (MONET Study) | Physical activity energy expenditure was not listed as a predictor for change in **IL-1β**, **IL-6**, **IL-8**, or **TNF-α** |
